# Public Attitudes Toward Pediatric Routine Immunization During the COVID-19 Pandemic in Saudi Society

**DOI:** 10.7759/cureus.17899

**Published:** 2021-09-12

**Authors:** Omaima A Alzaher, Ghada A Almutairi, Shahad M AlModhayan, Abdulaziz A Khojah, Ghaida A Almutairi

**Affiliations:** 1 Pediatric Medicine, Princess Nourah Bint Abdulrahman University, Riyadh, SAU; 2 Pediatrics, King Abdulla Bin Abdulaziz University Hospital, Riyadh, SAU; 3 Pediatric Medicine, Prince Sattam Bin Abdulaziz University, Al-Kharj, SAU

**Keywords:** routine vaccines, vaccination delay, saudi arabia, parents’ knowledge, parents’ attitude, immunization compliance, covid-19

## Abstract

Background

Routine vaccines are essential for the well-being of children. Due to the coronavirus disease 2019 (COVID-19) pandemic, some restrictions have been applied in the Kingdom of Saudi Arabia (KSA), causing parental concern about visiting healthcare facilities.

Objectives

To examine the attitudes and factors that influence parents’ decisions regarding their children's vaccination during the COVID-19 pandemic.

Methods

In this cross-sectional study, 1,704 caregivers from KSA participated. An implemented Arabic questionnaire directed to the caregivers of children at the age of routine immunizations was filled. It included questions on parent demographics, child data, the decision regarding the vaccines, cause-related questions, and the preferred means to receive the vaccines during the COVID-19 pandemic.

Results

A total of 1,360 participants were included. The majority were from the middle region of KSA. Thirty-nine point five percent (39.5%) of participants chose not to administer the scheduled vaccines to their children, and the main reason was the fear of COVID-19 infection. The main sources of information that encouraged administering the vaccines were the advice of physicians, Saudi Ministry of Health (SMOH) services, 937, and the Sehha application. The majority of the participants chose home immunization visits as the preferred means of vaccination during the COVID-19 pandemic (46.8%). The study showed that approximately 60% of the respondents believed that routine childhood immunizations had no role in spreading the COVID-19 infection, and 59% believed that despite the COVID-19 pandemic, routine childhood vaccines have to be administrated on time.

Conclusion

The study indicated the negative impact of the COVID-19 pandemic on the compliance of Saudi society toward routine pediatric immunization.

## Introduction

The coronavirus disease 2019 (COVID-19) pandemic is a crucial public health issue, as it grabs headlines worldwide. As a response to the pandemic, quarantine had started in the Kingdom of Saudi Arabia on March 23, 2020, and ended on June 21, 2020, while cautionary measures are still ongoing [1-2]. The Saudi Ministry of Health (SMOH) has established multiple applications to facilitate reaching health care services and ensure citizens' safety, and more means have been developed since the outbreak of the pandemic. The hotline 937 provides 24/7 call medical consultation services to fulfill citizens’ and residents’ health needs [3]. The application Sehha is another service that succeeded in covering all the Saudi cities, providing video calls, audio calls, and virtual medical consultations via chat. Both services continued helping to answer the population questions during the pandemic [4]. Mawid is another application that enables scheduling appointments across 2400 health care centers in the kingdom, and it sends repeated reminders [5]. When the COVID-19 pandemic started, an application called “Tetamman” (rest assured) was established. It aims to ensure safety and follow-up recovery for people in isolation or quarantine [6].

We recognize that the COVID-19 pandemic has many precedents and sequences and that it is disrupting life-saving immunization services globally, putting millions of children at risk of the targeted infections [7]. Numerous efforts of the Saudi government and initiatives by the MOH have been conducted to protect the community from illnesses by preserving children’s health, such as “vaccination reminder service,” a free SMS-based reminder that is sent to a phone or/and email week before the due date [8]. These worries about routine childhood immunization and the sequences are international. For example, the post of The Children’s Hospital of Philadelphia, based on CDC data, stated that more young children will be susceptible to measles this winter, a disease that approximately 1,300 people contracted last year. Although the social distancing that will probably be in place during the upcoming winter may lessen the risk of measles transmission, the fact that more children are now susceptible is worrisome. In addition, a recent study has illustrated that the uptake of vaccines in Europe has continued to decline in recent years, including in the United Kingdom [9]. The fall in measles, mumps, and rubella (MMR) vaccine coverage has been followed by large outbreaks of measles, with over 500,000 confirmed cases globally in 2019, more than in any single year since 2006. While spring and summer 2020 were anticipated to be accompanied by a lower incidence of many vaccine-preventable diseases, possible further declines in routine vaccine uptake in 2020 birth cohorts may surpass recent years, thus generating a record number of susceptible children.

If lockdown ends, social distancing relaxes, and formal schooling returns before, or during, 2020/21 autumn and winter season, then outbreaks of covered diseases appear inevitable [10]. As a preventive measure, on March 26, 2020, the WHO recommended the continuation of routine childhood immunization with temporary suspension of mass vaccination campaigns because they could spread coronavirus in the community [7].

Knowledge and attitudes toward routine immunization have been studied in different areas of the Kingdom of Saudi Arabia (KSA) before the COVID-19 pandemic in separate studies conducted in Najran, Majmaah, Jeddah, Madinah, and Hail. They revealed positive attitudes toward immunization (ranging between 78% and 92%) [11-17]. In this study, we aimed to emphasize the importance of routine childhood vaccination regardless of the pandemic, by assessing and describing the attitudes of Saudi parents toward these vaccinations during the pandemic. We also aimed to clarify the extent to which the pandemic has contributed to changing the rates of parental compliance and determine the factors that have led to decreased compliance or helped to maintain it. In addition, we aimed to recommend safe ways of vaccinations, to overcome people’s fears and minimize the risk of children or their parents being affected during this period and any similar situations in the future.

## Materials and methods

A cross-sectional, electronic, questionnaire-based study was conducted. The questionnaire was implemented using Google Forms. It contained five main items, including parent demographics, child information, cause-related questions, their source of information, and the preferred means of receiving the vaccines for both groups (those whose children received the vaccines and those whose children did not receive them during the COVID-19 pandemic). It was prepared in Arabic and English, after searching the available search engines using the keywords immunization, vaccine, attitude, knowledge, parents, and COVID-19 in different combinations.

The questionnaire was created and then reviewed by three native Arabic speakers’ faculties independently, to test their viability. To ensure validity, a pilot sample of 70 caregivers of the targeted population filled up the questionnaire, and feedback was provided. The questionnaire was modified, sent again, filled up, and finally re-revised. To prevent duplicates, an instruction to fill the questionnaire once by either parent (or caregiver) was written clearly on the front page and the Google Form was set not to accept more than one answer from each Google account. The Arabic questionnaire was sent electronically using email and social media platforms such as WhatsApp, Twitter, Telegram, and Facebook. A total of 1,704 caregivers’ responses were collected from June 3, 2020, to June 27, 2020. The study included different caregivers who had at least one child aged less than six years. After collecting the responses, the data were translated and coded in English in an Excel sheet (Microsoft Corporation, Redmond, WA) and followed by the exclusion process. The data were analyzed using the Statistical Package for the Social Sciences (SPSS) version 25 (IBM Corp., Armonk, NY). We described the qualitative data of age, gender, relationship to child, educational level, and nationality in frequencies and percentages. A chi-square test was performed to compare categorical variables, with P-value < .05 considered significant.

Before starting the data collection, the Princess Nourah Bint Abdulrahman University (PNU) Institutional Review Board approved the study protocol and the IRB log number is 20-0208. Online consents were obtained from the parents before filling the questionnaire to maintain the confidentiality of the data.

## Results

**Figure 1:**
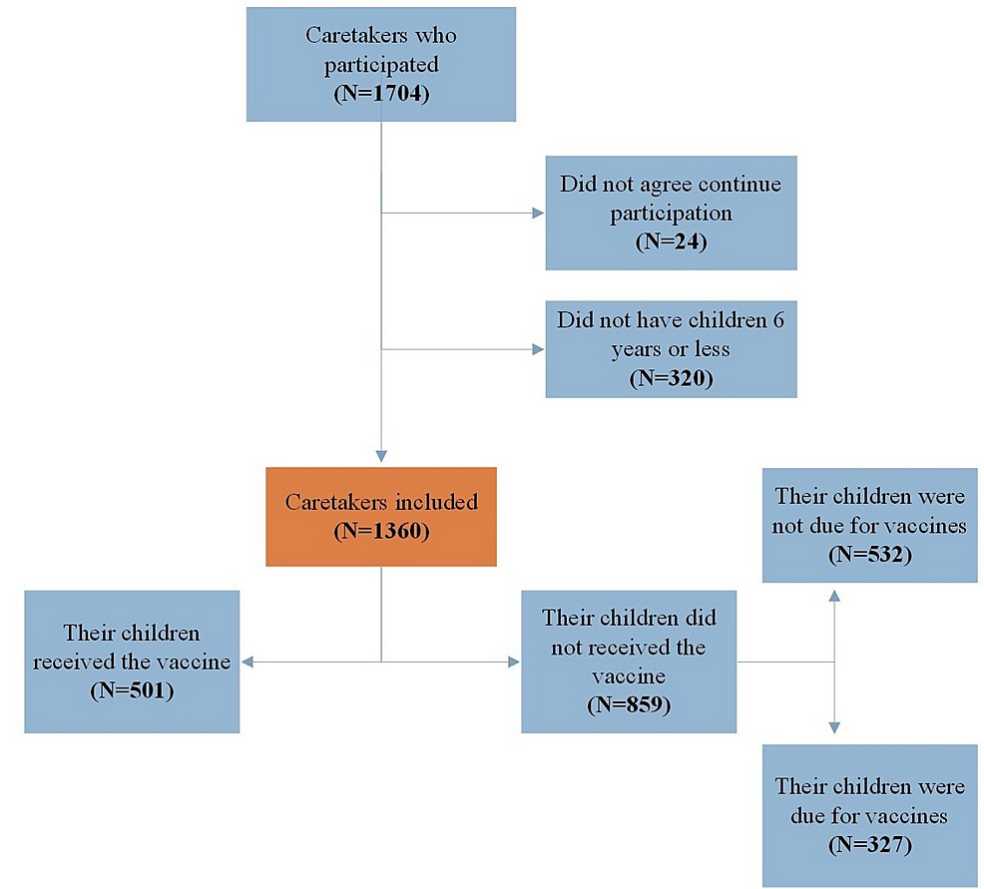
Data collection flowchart

Overall, 1,360 caregivers out of 1,704 were recruited after exclusion (Figure 1).

Table 1 shows the demographic data of the participants. The mothers were the majority of the respondents, more than two-thirds of the respondents were aged 40 years or less, and most of them were Saudi. The majority lived in the middle regions, were college graduates, or had higher education, and only 11.3% were working in the healthcare field. Most of the participants had one child of vaccination age during the COVID-19 pandemic, and in most cases, it was not the first child. The overall compliance rate was 60.5%.

**Table 1:** Participants’ socio-demographics (N = 1360)

Variable	Frequency (n)	Percentage (%)
Nationality		
Saudi	1275	93.8
Non-Saudi	85	6.3
Caretaker age (years)		
18-30	280	20.6
31-40	771	56.7
41-50	282	20.7
> 50	26	1.9
Relationship to the child		
Mother	1050	77.2
Father	234	17.2
Sibling	34	2.5
Other relative	42	3.1
Caretaker level of education		
None	17	1.3
Primary school	12	0.9
Intermediate or high school	173	12.7
College graduate	997	73.3
Higher education	161	11.8
Caretaker job		
Health care worker	153	11
Others	1207	89
Area of residence		
Middle	818	60.1
Eastern	163	12
Western	163	12
North	107	7.9
South	109	8
Number of children at immunization age		
One	1039	76
More than one	321	24
Does the immunization involve the first child?		
Yes	306	22.5
No	1054	77.5

**Figure 2:**
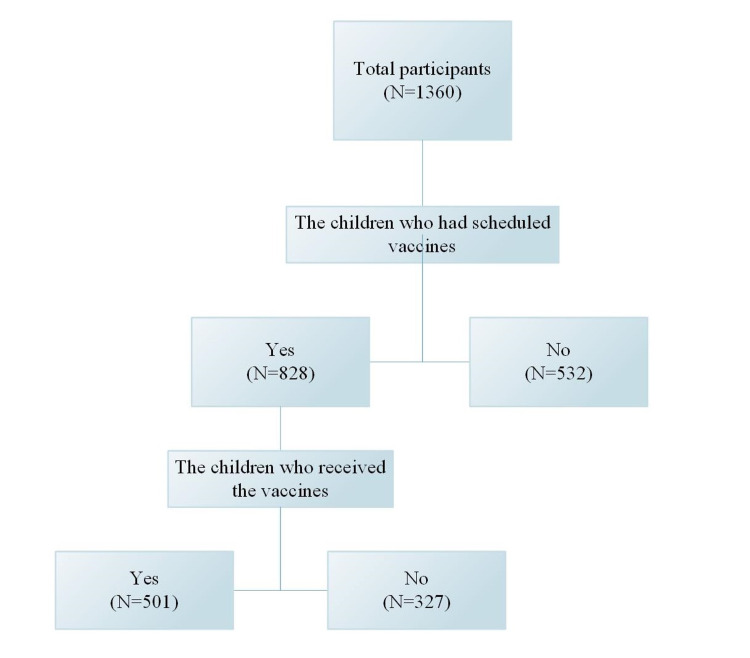
Flowchart of participants’ distribution

The total number of caregivers who answered that they did not administer the vaccines to their children was 63.16%; however, 61.9% of these children had no vaccines that were due during the pandemic period (Figure 2). Thus, only 327 participants (24% of the total and 39.5% of those whose children had vaccines that were due) decided not to administer the vaccines to their children, even though they were due. The majority of this group (n = 253, 77.4%) were afraid of COVID19 infection, whereas 86 participants (26.3%) could not reach the health care service, 47 participants (14.4%) were afraid of the side effects of the vaccines. A small number of participants answered that they thought the vaccines might cause diseases (n = 18, 5.5%), that the vaccines were not beneficial (n = 11, 3.4%), or that they preferred natural immunity (n = 5, 1.5%).

Although 532 participants had children who were not due for a vaccination, 19.2% were afraid of the risk of COVID-19 infection. Asking the group of participants who intended not to administer the routine childhood vaccines to their children regarding their sources of information revealed that 124 (37.9%) had no reference, whereas the most common source of information was personal experience or beliefs (n = 89, 27.2%). The other sources of information were health care workers (n = 41, 12.5%), family or friends’ experiences (n = 37, 11.3%), and the least common resources were researches that mentioned that the vaccines are harmful (n = 31, 9.5%).

When the group was asked about the factors that can contribute to changing their decision regarding immunization, the majority (n = 201, 61.5%) chose home visits for the vaccines as an alternative way to receive the vaccines, whereas physicians’ advice in a clinic visit represented a possibility of changing the decision for 93 (28.4%) participants. The lowest number (n = 55, 16.8%) chose the physician's advice on social media as a factor that could contribute to changing the decision. The majority of the group of caregivers whose children received their scheduled vaccines (n = 501) acquired their knowledge from health workers, such as pediatricians or family physicians in a direct conversation (n = 323, 64.4%), or using the ministry of health application “Sehha” and calling the hotline 937 (n = 147, 29.3%). Other resources included educational booklets (n = 130, 25.9%) and the Internet or social media (n = 105, 21%), whereas a small group (n = 18, 3.6%) had no reference. In addition, what drove them the most to seek vaccinations on time was the fear of the consequences of not receiving them (n = 3 12, 62.3%), the beliefs about their importance and significance in strengthening the immunity of the child against infections (n = 246, 49.1%), and the advice of health care workers (n = 168, 33.5%), whereas only nine (1.8%) respondents were encouraged by advice from friends or family members.

Considering demographic factors that may affect the compliance with vaccines in the population, there was no significant effect of the level of education, the job of the caretaker, having the first child due for a vaccine, or having one or more children. There were no significant differences in the age group distribution between those who administered the vaccine to their children and those who did not, except in the age group 41-50 years, as a higher percentage did not administer their children the vaccine (21% compared to 15.3% with P = 0.0352) (Table 2). Our study showed that almost 60% (n= 804) of the respondents believed that routine childhood immunization had no role in spreading COVID-19 infection.

**Table 2:** Comparing different factors affecting respondents who had scheduled immunization (those who gave and those who did not give a vaccine to their children) *Total number of participants who did not give their children scheduled vaccines = 327 †Total number of participants who gave their children the scheduled vaccines = 501 ‡P-value is significant if < 0.05

Factors	Did not give the vaccine*		Gave the vaccine†		Comparing the two groups	
	N	%	N	%	P-value‡	95% CI
Caregiver age group (years)						
18-30	72	22	124	24.8	0.3546	-3.189 to 8.539
31-40	183	56	288	57.5	0.6702	-5.353 to 8.390
41-50	68	21	77	15.3	0.0352	0.391 to 11.248
50>	4	1	12	2.4	0.1438	-0.654 to 3.261
Caregiver level of education						
None	3	1	7	1.4	0.6117	-1.523 to 1.996
Primary school	6	1.8	3	0.6	0.1017	-0.308 to 3.335
Intermediate or high school	43	13.1	60	12	0.6393	-3.405 to 5.925
College graduate	240	73.4	371	74	0.8480	-5.428 to 6.822
High education	35	10.7	60	12	0.5666	-3.297 to 5.577
Caretaker job						
Health care worker	38	11.6	58	11.6	1.000	-4.663 to 4.338
Other	289	88.4	443	88.4		
The vaccine was for the first child						
Yes	248	75.8	372	74.3	0.6268	-4.6185 to 7.3921
No	79	24.2	129	25.7		
Number of children at routine vaccination age						
1	234	71.6	368	73.45	0.5593	-4.2798 to 8.1511
More than 1	93	28.4	133	26.55		

The analysis revealed almost equally distributed percentages of those who did not have an opinion (n = 444, 32.6%), who agreed (n = 456, 33.5%), and who disagreed (n = 460, 33.8%) with the statement that there was no harm in delaying routine childhood immunization until the COVID-19 pandemic was over. Despite this, 806 respondents (59.3%) believed that despite the COVID-19 pandemic, routine childhood vaccines must be administrated on time. Table 3 shows the answers to the question about the preferred method of receiving or arranging routine childhood vaccines. The most frequent choice was home immunization visits as a preferred mode (n = 637, 46.8%).

**Table 3:** The caregivers preferred suggested ways for vaccinations during the COVID-19 pandemic N: number; % percentage

Method	N	%
Scheduled appointment in the primary health care	348	25.6
Visiting the primary health care center in their convenient time with no appointment	107	7.9
Home immunization visit	637	46.8
Delay the vaccination until the pandemic is over	268	19.7
Total	1360	100

## Discussion

Routine childhood immunization is a critical preventive measure against serious communicable infections. The Saudi MOH is dedicated to conducting such preventive measures using the timetable of routine childhood vaccines and campaigns based on the population requirements [18-20]. Worldwide, before the COVID-19 pandemic, more than 13 million children didn’t receive any vaccines.

During the pandemic, a decline in the ordered and administered pediatric vaccine has been reported in the United States, which can increase the risk of preventable diseases outbreak [21-22]. Another report has mentioned a significant drop in all vaccination in the immunization registry data with a 20% absenteeism rate for vaccinators [23].

In this study, despite the COVID-19 pandemic and lockdown orders, 60.5% of the participants who had children with routine childhood vaccines that were due, administered the vaccines to their children. A previous study conducted in 2018 at the National Guard Comprehensive Specialized Clinic in Riyadh, Saudi Arabia, targeting children aged 2-months to 6-years with delayed vaccination who attended the Well-Baby Clinic of the institute, showed that out of the 1000 children, 77.6% received a vaccine on time [24].

Another study conducted between May 2016 and August 2017 in Jeddah, Saudi Arabia, showed that delayed vaccinations were observed in 85/351 (24.2%) of the sample studied [25]. The rate compliance rate was lower in our study, and the primary cause for the delay was the fear of COVID-19 infection transmission (77.4%). Fear existed even among the parents of the children who did not have scheduled vaccines (19.2%), but there was a significant statistical difference between the incidence of fear between the two groups (P-value < 0.0001). These results indicate the decline in compliance as reported worldwide, which is creating a real fear for the future of spread of the preventable disease. The availability of home immunization visits was the most common choice (n = 201, 61.5%) for changing the attitude toward vaccination in this group. This again proves that the safety of their children during the spread of COVID-19 infection is their main concern and the reason for their negative attitude toward routine immunizations.

The main references for information for the compliant group were specialized physicians (n=176, 35.4%), and the MOH services, such as the 937 hotline and application “Sehha” (n=142, 29.3%). We think people should be more aware of utilizing the mentioned services, especially when they do not have a direct access to health care workers to answer their inquiries. A high percentage of the respondents (59%) believed that despite the COVID-19 pandemic, routine childhood vaccines must be administered on time. Although it is a lower percentage in comparison with the previous studies that investigated the awareness level before the pandemic, as they determined a positive attitude toward immunization, ranging between 78% and 92%, it still reflects a good understanding of the importance of vaccines and a positive attitude [11-17].

Furthermore, 33.5% of respondents disagreed and 33.8% agreed with the statement that there was no harm in delaying routine immunization until the COVID-19 pandemic was over, which implies that the issue is confusing to the caregivers. Therefore, more emphasis on sticking to routine vaccine timetables is needed. These will be more effective if they come from specialized health care workers, as the results showed that they were the main source of information for those who adhered to the vaccination schedule.

To conclude with suggestions that can contribute to improving the compliance during quarantine situations, a question has been directed to the participants about the preferred means to arrange or receive the routine vaccination service that already exists for free to all the citizens in Saudi Arabia via the primary health care centers [18]. Approximately half of the participants preferred home immunization visits, which highlights the importance of providing safe alternative strategies during quarantine situations. One-fifth still preferred delaying the vaccines until the pandemic was over, which may illustrate the existing fears regarding COVID-19 infection.

Our study has the following limitations. It was conducted during the COVID-19 pandemic lockdown. Therefore, the online questionnaire was distributed through emails and social media as the whole population of Saudi Arabia was under community-containment measures and asked to be home confined unless necessary even for the hospital visits. To minimize the impact of this on the results, the questionnaire was validated, and a pilot test was conducted.

Moreover, 39% of the participants who had children at the age of routine immunization had no due vaccines, which reduced the input on some of the questions and might have played a role in the result of the calculated rate of compliance. The unequal representation of Saudi Arabian regions is another limiting factor; however, the actual difference in the population distribution between these regions must be considered.

## Conclusions

The present study demonstrated the negative impact of the COVID-19 pandemic on Saudi society's attitude toward pediatric routine immunization, with the main concern being the fear of COVID-19 infection. With this impact, nearly 59.3% accepted that the scheduled childhood immunizations had no role in spreading COVID-19 infection and 59.1% agreed that despite the COVID-19 pandemic, scheduled childhood immunizations must be administrated on time, which demonstrates a good understanding of the importance of vaccines. The largest group of respondents (46.8%) preferred home immunization services for vaccinations during the COVID-19 pandemic, which highlights the importance of providing safe alternative strategies during quarantine situations. We strongly recommend modifying routine immunization programs during special situations such as a quarantine. A possible modification is adopting home vaccination programs, if feasible, to improve compliance and reduce the adverse consequences of delaying or skipping routine immunizations.

In addition, developing official online or telecommunication modalities with the parents of children scheduled for vaccination to answer their inquiries with good announcements about the services is recommended. Utilizing social media for announcements is also encouraged under the supervision of health care personnel, as the impact of both on our sample was noted. Increased utilization of the existing services provided by the Saudi MOH, such as the 937 hotline and Sehha application, is recommended; it requires means to increase the awareness of the population about these services. Lastly, we suggest appointing pediatricians or family physicians in primary care services to address parents' concerns regarding vaccinations.
